# Effects of partial replacement of red by green light in the growth spectrum on photomorphogenesis and photosynthesis in tomato plants

**DOI:** 10.1007/s11120-021-00879-3

**Published:** 2021-09-27

**Authors:** Magdalena Trojak, Ernest Skowron, Tomasz Sobala, Maciej Kocurek, Jan Pałyga

**Affiliations:** 1 Department of Medical Biology, Jan Kochanowski University, Uniwersytecka 7, 25-406 Kielce, Poland; 2 Department of Environmental Biology, Jan Kochanowski University, Uniwersytecka 7, 25-406 Kielce, Poland

**Keywords:** Green light, Photomorphogenesis, Photosynthesis, Shade-avoidance syndrome, Spectrum optimization

## Abstract

The artificial light used in growth chambers is usually devoid of green (G) light, which is considered to be less photosynthetically efficient than blue (B) or red (R) light. To verify the role of G light supplementation in the spectrum, we modified the RB spectrum by progressively replacing R light with an equal amount of G light. The tomato plants were cultivated under 100 µmol m^–2^ s^–1^ of five different combinations of R (35–75%) and G light (0–40%) in the presence of a fixed proportion of B light (25%) provided by light-emitting diodes (LEDs). Substituting G light for R altered the plant’s morphology and partitioning of biomass. We observed a decrease in the dry biomass of leaves, which was associated with increased biomass accumulation and the length of the roots. Moreover, plants previously grown under the RGB spectrum more efficiently utilized the B light that was applied to assess the effective quantum yield of photosystem II, as well as the G light when estimated with CO_2_ fixation using RB + G light-response curves. At the same time, the inclusion of G light in the growth spectrum reduced stomatal conductance (*g*_s_), transpiration (*E*) and altered stomatal traits, thus improving water-use efficiency. Besides this, the increasing contribution of G light in place of R light in the growth spectrum resulted in the progressive accumulation of phytochrome interacting factor 5, along with a lowered level of chalcone synthase and anthocyanins. However, the plants grown at 40% G light exhibited a decreased net photosynthetic rate (*P*_n_), and consequently, a reduced dry biomass accumulation, accompanied by morphological and molecular traits related to shade-avoidance syndrome.

**Supplementary Information:**

The online version contains supplementary material available at 10.1007/s11120-021-00879-3.

## Introduction

The light environment strongly influences plant development and physiology because the broad-spectrum of incident radiation absorbed by different groups of photoreceptors represents a signal for light-induced photomorphogenesis (Paik and Huq 2019). Phytochromes (PHYs) that regulate seed germination, de-etiolation, shade-avoidance syndrome (SAS) responses, circadian clock and blooming are the most sensitive to red (R) and far-red (FR) radiation. UV-A, blue (B) and green (G) light receptive cryptochromes (CRYs) are essential to the regulation of de-etiolation, entrainment of the circadian clock and flowering. Phototropins that absorb UV-A, B and G light regulate phototropism, including hypocotyl and stem bending, leaf positioning, as well as chloroplast movements (Casal 2013; Cope et al. 2014; Huché-Thélier et al. 2016). Photosynthesis, however, is driven by the absorption of light via chlorophylls and auxiliary pigments, such as carotenoids. The wavelengths of light that drive photosynthesis are referred to as photosynthetically active radiation (PAR) and range from 400 to 700 nm. Due to different absorption rates, not all wavelengths are equally efficient in carbon dioxide assimilation (Cope et al. 2014).

The effects of light with different spectral qualities on plant growth and development are not fully understood because the action of the monochromatic light applied for photosynthesis and photomorphogenesis analyses (Izzo et al. 2020) are not strictly reproduced when added to a mixed spectrum of different wavelengths (Kaiser et al. 2019). Moreover, most studies have focussed on R and B light (Bantis et al. 2018), whilst the light wavelengths, such as G and FR, have been traditionally considered to be less or even inactive for photosynthesis, and are thus excluded from the spectrum of artificial lighting systems. However, the latest reports by Bian et al. (2018a), Bian et al. (2019) and Kaiser et al. (2019) appear to suggest that the influence of G light on plants deserves more research.

The reason why RGB spectrum is less commonly used than RB is the fact that the fraction of incident light quanta successfully utilized by plants to drive electron transport is approximately 20% lower for G light than for B light (Sun et al. 1998) and depends on the leaf’s internal structure and thickness, as well as the content of photosynthetic pigments (Falcioni et al. 2017). Secondly, the weak absorption of leaf extracted chlorophylls *a* and *b* in the G light range contributes to the misconception that green light is only poorly absorbed by plant leaves, in contrast to blue and red light (Karlický et al. 2021). Terashima et al. (2009) demonstrated, however, that the addition of G light to the white (W) light with high photosynthetic photon flux density (PPFD) is more effective in driving photosynthesis than additional R or B light. This is because the chlorophylls in palisade cells, which absorb the majority of the R and B of the incident light, allow G light to penetrate deeper into the leaf in order to provide energy for light reactions in the chloroplasts located in the spongy mesophyll cells (Terashima et al. 2011). The G light-dispersion inside the leaf blade causes the so-called ‘détour effect’ (Terashima et al. 2009; Kume 2017; Smith et al. 2017), which provides the photons the chance to hit and be absorbed by the chloroplasts that are even located close to the abaxial surface of the leaf. Consequently, the G light absorbed by the leaf can drive photosynthesis, with an overall quantum efficiency greater than that of B light and equal to that of R light (Hogewoning et al. 2012; Smith et al. 2017). Thereby plants possess adaptations to perceive and utilize green light, especially under the canopy of leaves, where transmitted light is depleted in the red and blue part of the spectrum (Matthews et al. 2020; see Appendix, Fig. S1) absorbed primarily by chlorophyll of upper leaves (de Wit et al. 2016).

As suggested earlier (Bantis et al. 2018; Kaiser et al. 2019), G light promotes plant growth by increasing the leaf area and because of the fresh and dry masses of the plants. It is also involved in seed germination and flowering and is responsible for SAS by modifying the rate of stem and petiole growth in order to compete for light availability in a dense phytocoenosis (Zhang et al. 2011). Moreover, recent reports (Bian et al. 2018a, b) have also indicated the beneficial effects of the supplementation of RB spectrum with low G light intensities (approximately 60 µmol m^–2^ s^–1^), including higher photosynthetic efficiency, improved antioxidative properties and the enhanced activity of nitrogen assimilation enzymes.

Tomato is an important horticultural crop (Schwarz et al. 2014), but it is often cultivated under inadequate light conditions when grown in an indoor system (Yan et al. 2018). Thus, the objective of the present study is to investigate the effects of partial red-to-green light replacement in the RB-LED spectrum on the tomato plants’ physiological activity and photomorphogenesis. To this end, we first examined the morphological features and stomatal traits in the tomato plants grown under different light spectra. Secondly, we determined whether supplementation of low-intensity RB spectrum with the variable doses of G light in place of R could improve the photochemical activity of photosystem II, CO_2_ assimilation and water-use efficiency. Thirdly, we have tested the effects of growth under different light spectra with low PPFD on the subsequent photosynthetic utilization of light with higher PPFD. We have also attempted to distinguish between SAS responses induced by decreased R light intensity or increased G light contribution by analysing the levels of phytochrome interacting factor 5 (PIF5), chalcone synthase (CHS) and anthocyanins. This allowed us to establish how the plant responds to the G light-enriched RB spectrum in order to promote photosynthesis and to restrain the shade-avoidance reactions in response to reduced R light intensity, especially under low light conditions. Moreover, as the previous research on SAS has usually focussed on the changes in the R/FR light ratio, thus ignoring the importance of the G/B ratio as a signal that also activates plant responses to canopy shade (Sessa et al. 2018), we tested a progressive substitution of R by G light in the fixed proportion of B light (25%). Our results suggest that a 20–30% contribution of green light in the RGB spectrum should be considered for indoor cultivation at a low light intensity in order to improve the photosynthetic activity and water-use efficiency of plants.

## Materials and methods

### Plant material and light treatment

Tomato (*Solanum lycopersicum* L. cv. Malinowy Ozarowski) seeds, treated with antifungal powder (T75 DS/WS), were germinated in Petri dishes on sterile filter papers soaked in Milli-Q-water for 1 day at 26 °C. Analysed tomato cultivar is a potato leaf phenotype that showed reduced leaf dissection (Busch et al. 2011). After germination, tomato seedlings with similar root lengths were placed into plastic pots containing substrate (white and black peat, perlite and N: P: K = 9: 5: 10; pH 6.0–6.5) and were grown under a white LED light (approximately 100 µmol m^–2^ s^–1^). Seven days after sowing, the seedlings were transplanted to P9 containers (9 × 9 × 10 cm) and were filled with the same substrate, divided into groups and transferred to five growth chambers, with no-reflected black separators in order to eliminate light contamination. Plants were grown under Px256 PxCrop LED lamps (PXM, Podleze, Poland) and were equipped with 9 modules composed of 4 individual LEDs (2 × R, 1 × G, 1 × B) each. This allowed us to achieve a very accurate mixing of light to illuminate the plants. The plants were grown for 24 consecutive days under LED modules that delivered 100 µmol m^–2^ s^–1^ PPFD (C, G10, G20, G30 and G40) with different light spectra: red-LEDs [peak wavelength: 671 nm, peak broadness at half peak height: 25 nm (656–681 nm)]; green LEDs (524 nm/40 nm/505–545 nm) and blue LEDs (438 nm/20 nm/428–448 nm). The C plants were used as the control group for G10–G40 plants (GX plants). Light spectra were determined with a calibrated spectroradiometer GL SPECTIS 5.0 Touch (GL Optic Lichtmesstechnik GmbH, Weilheim/Teck, Germany). The G and R contribution to a constant (25%) B light PPFD was denoted as G/B and R/B, respectively. The R/FR ratio was assessed by the analysis of the R-LEDs emission spectrum, as no additional FR-LEDs were used in the study. The light spectra of growth chambers are designated in Fig. S2, and the details of light treatments are summarized in Table 1.

**Table 1:** Description of the spectral composition used in the study and the ratio of photon flux integral (µmol m^–2^ s^–1^) of R and FR (R/FR), G and B (G/B), R and B (R/B) radiation

Treatment	Spectral characteristics of lighting treatments						
	% R^a^	% G	% B	FR^b^	R/FR	G/B	R/B
C	75	0	25	0.20	344.8	–	3.0
G10	65	10	25	0.17	340.3	0.4	2.6
G20	55	20	25	0.05	1011.9	0.8	2.2
G30	45	30	25	0.05	958.2	1.2	1.8
G40	35	40	25	0.02	2004.9	1.6	1.4

The light spectra of growth chambers were recorded with a spectroradiometer at six locations at the level of the apical bud and averaged. Plants of all groups were grown under 100 µmol m^–2^ s^–1^ PPFD. C states for the control plants (75R:25B). The rest of the plants (G10–G40) were grown under the RGB spectrum provided by progressive replacing from 10% (G10) to 40% (G40) of R light with an equal amount of G light

^a^Percentage of R (601–700 nm), G (501–600 nm) and B (401–500 nm) radiation of total (100 µmol m^–2^ s^–1^) PPFD (400–700 nm)

^b^Photon flux integral of FR (701–750 nm) radiation in µmol m^–2^ s^–1^

The plants were watered with tap water when necessary and fertilized once a week with 1% (w*/*v) tomato fertilizer (N: P: K = 9: 9: 27; Substral Scotts, Poland). The light spectra composition and PPFD were monitored daily by a spectroradiometer, and the readings were averaged for six locations at the level of the apical bud and maintained at 100 μmol m^−2^ s^−1^ by adjusting the distance between the light sources and the plant canopies. The containers with tomato plants cultivated under the same light illumination were turned away twice a day. To avoid canopy shading and overlapping, five plants per square metre of the illuminated area were cultivated. A photoperiod was 16/8 h day/night (day 6:00 am–10:00 pm), the average temperature was maintained at 25/22 °C day/night and relative air humidity was kept at 50–60%. The second leaf from above the 24-days-after-transplanting (DAT) tomato plants was used for all subsequent analyses. All analyses were conducted between 8:00 am and 12:00 pm. 30 tomato plants (three repetitions with ten plants per light treatment) were grown with each kind of light composition.

### Morphological analyses

The changes in plant height were measured at 3-day intervals and stem length was measured from the shoot apex to the base. The internode and petiole length, as well as leaf area, were assessed using high-resolution scans analysed with AxioVision 4.8 software (Carl Zeiss Inc., Oberkochen, Germany). Roots were gently washed after removing the substrate and scanned. The scans were analysed using ARIA v.2.0 software (Iowa State University, USA) (Pace et al. 2014). The total root length represents the cumulative length of all roots. The leaf inclination angle, which is the angle of the leaf above the horizontal with the base of the petiole at the vertex (see Appendix Fig. S4), was measured according to the work by Mullen et al. (2006). After non-invasive measurements, plants from each group were dissected into root, stem and leaves (with petioles) using a sharp scalpel. For dry biomass determination (DM), the collected material was dried at 105 °C for 24 h and weighed. Ten plants (two plants per replicate and five replicates per light treatment) were randomly selected for each determination.

### Stomatal traits analyses

The leaf epidermal strips from 24-DAT plants were used for all morphological stomatal features. Ten randomly selected leaves (one leaf per plant) per light treatment were collected in the morning and the epidermal strips (four strips per leaf) were peeled off from the abaxial side of the leaf (avoiding leaf veins) and were allowed to float on 2 ml of a basal reaction mixture (5 mM (2-(N-morpholino)ethanesulfonic acid, MES), 50 mM KCl, 0.1 mM CaCl_2_, pH 6.5) for 2 h (Wang et al. 2010). Stomatal traits were analysed with the images obtained by a Nikon Eclipse E100 microscope with AxioVision 4.8. For the individual stomatal traits, a magnification of × 1000 was used and ten randomly selected stomata per sampling area were measured. The stomatal width, stomatal length, pore width (minor axis of the pore) and pore length (major axis of the pore) were measured. For the width/length ratio, stomata width, including pore width, was chosen instead of the guard cell width, since the latter changes as the stomata close. The stomatal pore area (µm^2^) was also determined using AxioVision 4.8. Stomatal density (*S*_d_) was determined under a magnification of × 250 with five different fields of view per sampling area. Pore area per leaf area was calculated as the ratio of a cumulative pore area of the stomata (based on *S*_d_) to the abaxial leaf surface (Savvides et al. 2011).

### Chlorophyll, carotenoids and anthocyanins assays

The concentrations of chlorophyll *a* and *b* (Chl *a*, *b*) and total carotenoids were measured spectrophotometrically with UV VIS Helios Gamma (Thermo Spectronic, Waltham, USA) after being dissolved in dimethyl sulfoxide (DMSO). Pigments were extracted from leaf discs (3 mm in diameter, approximately 20 mg of tissue, one disc per leaf, ten leaves per light treatment) in 1.5 ml DMSO per leaf disc. Samples, kept in dim light, were vortexed for 1 min, capped and incubated for 3 h at 65 °C with inversion every 10 min to improve extraction. Then, the sample mixture was centrifuged at 10,000 × *g* for 15 min, and then the supernatant was carefully collected without disturbing the plant tissue, transferred to a new tube and mixed once again for 15 s. An aliquot (1 ml) of the uppermost supernatant layer was used for pigment determination at 480, 649 and 665 nm, according to the optimized method described by Wellburn (1994). The levels of anthocyanins were measured, as described by Laby et al. (2000). Plant tissue (200 mg) was extracted with 1 ml methanol: HCl (99: 1, v/v) at 4 °C. The samples absorbance (A) was spectrophotometrically measured at 530 and 657 nm, and the relative anthocyanins levels were determined using Eq. 1:

1 $${\text{A}}_{{{53}0}} \,{-}\,\frac{{\left( {0.{25}\, \times \,{\text{A}}_{{{657}}} } \right)\, \times \,{\text{Extraction}}\,{\text{volume}}\,\left( {{\text{ml}}} \right)\, \times \,{1}}}{{{\text{Mass}}\,{\text{of}}\,{\text{tissue}}\,{\text{sample}}\,\left( {\text{g}} \right)}}\, = \,\frac{{{\text{Relative}}\,{\text{units}}\,{\text{of}}\,{\text{anthocyanins}}}}{{{\text{g}}\,{\text{Fresh}}\,{\text{mass}}\,{\text{of}}\,{\text{plant}}\,{\text{tissue}}\,\left( {{\text{AU}}\,{\text{g}}^{{{-}{1}}} \,{\text{FM}}} \right)}}.$$

The analysis of each light treatment was replicated ten times with one leaf per replicate.

### Determination of photochemical reflectance index (PRI)

The PRI was determined from the reflectance (*Ref*) of the tomato’s leaf and was measured directly using a spectroradiometer GL SPECTIS 5.0 Touch, attached via an optical fibre to the externally integrated LI-1800-12S sphere (LI-COR Inc., Lincoln, USA) with a tungsten halogen lamp emitting a broad-spectrum of 380–2500 nm, calibrated with a standard magnesium oxide dish with a reflectance assumed to be 100%. The leaf samples were irradiated with the chamber-specific light just before PRI determination (the time gap between the PRI measurement and the irradiation was below 1 min), as was proposed by Kohzuma and Hikosaka (2018). PRI was calculated as follows (Eq. 2):

2 $${\text{PRI}}\, = \,\frac{{\left( {Ref_{{{531}}} \,{-}\,Ref_{{{57}0}} } \right)}}{{\left( {Ref_{{{531}}} \, + \,Ref_{{{57}0}} } \right)}},$$

where *Ref*_531_ is the reflectance at 531 nm (xanthophyll signal) and *Ref*_570_ represents reflectance at 570 nm (a reference waveband) (Gamon et al. 1997). Each treatment consisted of ten replicates per light treatment with two leaves per replicate.

### Measurement of chlorophyll fluorescence (ChF) induction kinetics

ChF induction kinetics was performed using a pulse amplitude modulated (PAM) fluorometer (Maxi IMAGING PAM M-Series, Walz, Effeltrich, Germany). The minimal fluorescence level (Fo) with all PSII reaction centres open was measured using the measuring modulated blue light (450 nm), which was sufficiently low (0.01 µmol m^–2^ s^–1^) in order to not induce any significant variable fluorescence. The maximal fluorescence level (Fm) with all PSII reaction centres closed was determined by a 0.8 s saturating pulse at 2700 µmol m^–2^ s^–1^ in 30 min dark-adapted leaves at room temperature (RT). Then, leaves were continuously illuminated with a low intensity of blue actinic light (AL, 110 µmol m^–2^ s^–1^) to match the flux of the growth light. After about 4 min, the steady-state value of fluorescence (Fs) was thereafter recorded and a second saturating pulse at 2700 µmol m^–2^ s^–1^ was imposed to determine the maximal fluorescence level in the light-adapted state (Fm’). The maximum quantum yield of PSII (Fv/Fm), the effective quantum yield of PSII photochemistry (*Φ*_PSII_), the quantum yield of regulated energy dissipation in PSII (*Φ*_NPQ_) and the non-regulated energy dissipation in PSII (*Φ*_NO_) were measured.

The excitation pressure on PSII, reflecting the ratio of reduced to the overall pool of the primary acceptor of PSII (Q_A_), was estimated with AL = 186 µmol m^–2^ s^–1^, based on the light-response curve (data not shown) in order to match the highest value of the relative PSII electron transport rate (ETR), and low enough to avoid photoinhibition (Dahal et al. 2017). ETR (Eq. 3) was calculated based on the effective PSII quantum yield (measured at 186 µmol m^–2^ s^–1^), the incident photon flux density (PFD = 186 µmol m^–2^ s^–1^) and the PFD-Absorptivity (Abs.). Absorptivity measures the fraction of the incident light absorbed by the leaf sample first illuminated with red (R) and then with near-infrared (NIR) light (Eq. 4). The partitioning of the absorbed light between PSI and PSII was assumed to be equal (each photosystem receiving 0.5) (Oguchi et al. 2021). Each measurement comprised six replicates per light treatment with four leaves per replicate.

3 $${\text{ETR}}\, = \,0.5\, \times \,\Phi_{{{\text{PSII}}}} \, \times \,{\text{PFD}}\, \times \,{\text{Abs}},$$

4 $${\text{Abs}}.\, = \,\frac{{{1}\,{-}\,{\text{R}}}}{{{\text{NIR}}}}.$$

### Leaf gas exchange measurements and the quantum yield of the G light

The photosynthetic parameters were measured using a Li-6400XT Portable Photosynthesis System (LI-COR Inc.) with the 2 × 3-cm transparent chamber (6400-08) illuminated with the RGB-LED Light Source SL 3500-C (Photon Systems Instruments, Brno, Czech Republic) or directly in chambers (in situ) with a group-specific light composition. Each measurement was comprised of four replicates with four leaves per replicate. The leaf cuvette conditions were maintained at a relative air humidity of 60%, air temperature in cuvette set to constant 25 °C, 400 µmol mol^–1^ of external CO_2_ concentration and gas flow rate of 500 ± 2 µmol s^–1^. The values of the net photosynthetic rate (*P*_n_), stomatal conductance (*g*_s_), intercellular carbon dioxide concentration (*C*_i_), transpiration rate (*E*) were recorded in situ within about 300 s per replicate to stabilize gas exchange. For water-use efficiency (WUE) assessment, the intrinsic (*WUE*_int_; *P*_n_/*g*_s_) and instantaneous water-use efficiency (*WUE*_ins_; *P*_n_/*E*) were estimated (Medrano et al. 2015). Moreover, vapour pressure deficit (*VPD*) and leaf temperature *(T*_leaf_) were analysed. Then, the RB + G light-response curves (RB + G_curve_) were measured using variable doses of G light (0–450 µmol m^–2^ s^–1^; G: 530 nm) at a constant RB background (100 µmol m^–2^ s^–1^; 75R:25B; R: 627 nm, B: 447 nm) plotted up to 550 µmol m^–2^ s^–1^ and limited by the SL 3500-C output power of G-LEDs. The additional G light was applied at the following steps: 0, 50, 100, 200, 300, 400 and 450 μmol m^–2^ s^–1^. For the light-response curve following steps were time-separated (200 s) to stabilize gas exchange.

Besides, based on Terashima et al. (2009) and Paradiso et al. (2011), we also tested the quantum yield of the G light (*Φ*_530_), defined as the ratio of the CO_2_ fixation rate per absorbed green light intensities (0–450 μmol m^–2^ s^–1^) and fitted with a non-rectangular hyperbola using the non-linear fitting procedure (NLIN) (Hogewoning et al. 2010) in STATISTICA 13.3 software (TIBCO Software Inc., Palo Alto, CA, USA) (Table 4). The absorptance of G light (530 nm) was based on the leaf optical properties measured with spectroradiometer GL SPECTIS 5.0 Touch, attached via an optical fibre to the externally integrated LI-1800-12S sphere (LI-COR Inc.) with a tungsten halogen lamp emitting a broad-spectrum of 380–2500 nm (Table 4).

### Leaf protein extraction and preparation—analysis of Rubisco content

Soluble leaf proteins (SLP) were extracted with a Plant Total Protein Extraction Kit (Sigma-Aldrich, St. Louis, USA) according to the manufacturer’s instruction and the extract was employed for either the quantification of the amount of Rubisco enzyme or for the detection of a specific protein using the western blot (WB) procedure. In brief, twelve randomly selected fresh leaves of each light treatment (three leaves per replicate) were immediately frozen in liquid nitrogen and grounded separately using a chilled mortar and pestle with liquid nitrogen. Then 200 mg of leaf powder per replicate, protected from proteolysis by a protease inhibitor cocktail, was washed with a methanol working solution and acetone. A purified tissue pellet was used for total protein extraction with a chaotropic protein reagent. For the PIF5 protein analysis, the collected leaves were kept in the dark, and then protein extraction was performed under as little light as possible, as described by Shen et al. (2007). The soluble protein content was estimated according to Bradford’s work (1976), using Coomassie reagent (Thermo Fisher Scientific, Waltham, USA) and bovine serum albumin as a standard. The aliquots containing 5 µg protein per lane were loaded onto precast 4–20% gradient TGX polyacrylamide gel (Bio-Rad, Hercules, USA). The integrity of the protein resolved was analysed with Bio-Safe™ Coomassie Stain (Bio-Rad). The Coomassie-stained protein bands have also been used for the quantification of RbcL (the large) and RbcS (the small subunit of ribulose-1,5-bisphosphate carboxylase/oxygenase) carried out using densitometric analysis (ImageJ v.1.49, National Institutes of Health, Maryland, USA) (Tercé‐Laforgue et al. 2004). Then, the relative amount of protein of interest was assessed based on the assumption that the densitometric value recorder for C plants is unity (100%). The samples were analysed four times per light treatment.

### Western blot analysis

Optimized amounts of extracted proteins were loaded onto precast 4–20% gradient TGX polyacrylamide gels (Bio-Rad) and were run with a constant voltage of 200 V for 20 min. Separated proteins were transferred to nitrocellulose membranes (0.45 µm or 0.2 µm pore size; Bio-Rad) by semi-dry electroblotting (1.5 mA per cm^2^, 20 min). Air-dried blots were blocked with 5% non-fat dry milk blocking reagent (1 h, RT) (Bio-Rad) and were incubated with primary antibodies against RCA (ribulose-1,5-bisphosphate carboxylase/oxygenase activase; AS10 700; 1:5000), PIF5 (phytochrome interacting factor 5; AS12 2112; 1:1000) or CHS (chalcone synthase; AS12 2615; 1:1000) and ATPB (beta subunit of ATP synthase; AS05 085; 1:5000) (Agrisera, Vännäs, Sweden) overnight at 4 °C. Then, membranes were washed in Tween-TBS buffer (0.05% Tween 20, 20 mM Tris, 500 mM NaCl, TTBS) and incubated with a horseradish peroxidase-conjugated secondary antibody (AS09 602; 1:5000–1:10,000) for 1 h at RT with agitation. The blots were washed in TTBS and developed for 5–10 min with a colorimetric detection reagent using Pierce™ DAB Substrate Kit (3,3′-diaminobenzidine tetrahydrochloride; Thermo Fisher Scientific). The quantification of the protein bands of the WB membranes visualized with DAB was made using densitometric analysis (ImageJ v.1.49) (Tercé‐Laforgue et al. 2004). To investigate PIF5 and CHS proteins, 60 µg of total protein was separated in the gels and a higher concentration of the detection reagent was used. For RCA and ATPB, 5 µg of the total protein per lane was separated in the gels. The ATPB was used as a loading control. Then, the relative amount of protein of interest was assessed based on the assumption that the densitometric value recorder for C plants is unity (100%). The samples were analysed four times per light treatment.

### Statistical analysis

The normal distribution of variables was verified using the Shapiro–Wilk’s test and the equality of variances was evaluated using Levene’s test. The one-way ANOVA and post hoc Tukey’s HSD tests were employed to analyse the differences between the investigated groups. Pearson correlation analysis (*r*) was conducted to measure the relevance between parameters. Statistical significance of all analyses was determined at the 0.05 level (*p* = 0.05). The data are presented as the mean with a standard deviation (± SD). Statistical analyses were performed using STATISTICA 13.3 software (TIBCO Software Inc.).

## Results

### The effect of light quality on plant morphology and biomass accumulation

**Fig. 1:**
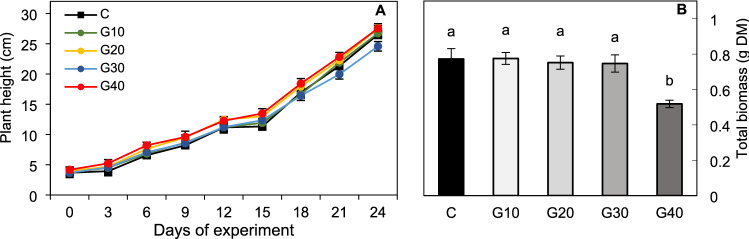
**A** Plant height (cm) measured at three-day intervals up to 24-DAT and **B** total dry biomass (DM) (leaves with petioles, stem and root) on 24-DAT in tomato plants grown under different light conditions. The values are means of ten replicates ± SD. Different letters (*a*, *b*) indicate significant differences between treatments at *p* = 0.05 with a Tukey’s HSD test

The height of the tomato plants was measured at three-day intervals following seedling transplantation to chambers with a different light spectrum (Fig. 1A, Day 0). On the 24th day of the following transplantation, no differences in the height between G10, G20 and G40, and the control (C) plants were observed (Table 2; Fig. S4). Only the 24-DAT G30 plants showed a 7% deceleration in the growth rate compared to C (Table 2; Fig. S4). At the same time, the total dry biomass (Fig. 1B) of G10–G30 plants, as well as the dry mass of the stem of G20 and G30 plants (Table 2), were indistinguishable from those noted under RB light. Meanwhile, G10 plants presented increased, whilst G40 plants decreased the dry biomass of the stem. Moreover, the G10, G20 and G40 plants produced noticeably longer petioles than the C plants, and those in the G20–G40 groups also exhibited increased leaf inclination angles, whilst the G40 plants also showed a tendency to reduce the leaf area (Table 2; Fig. S3B) and significantly reduced the total dry biomass (Fig. 1B). Overall, substituting R radiation with G altered the biomass partitioning to the leaves and roots in opposite ways. Observed decreased shoot-to-root dry mass ratio within G10–G30 plants (Table 2) along with unchanged total dry biomass proved that plants allocated more assimilates to the underground (roots) than aboveground (shoot = stem + leaves with petioles) structures than those grown under RB light only. We noticed especially, that increasing G light contribution decreased the dry mass of leaves by 25%, 13% and 41% in G10, G20 and G40 plants, respectively. The opposite reaction was observed for roots: G light supplementation at 10–30% increased roots dry mass by 33% to 14% (Table 2). Consequently, G10, G20 and G30 plants were characterized by strongly developed root structures (Fig. S3C), with a significant increase in total root length (Table 2), compared to control. However like in C plants, also in G40 group, the root system was poorly expanded, and its dry mass and total root length were significantly lower than noted for other plants grown under RGB light (Table 2).

**Table 2:** Morphological and stomatal traits of 24-DAT tomato plants (*Solanum lycopersicum* L. cv. Malinowy Ozarowski) grown under different light conditions

Parameter	Treatment				
	C	G10	G20	G30	G40
Morphological traits					
Height of 24-DAT plants (cm)	26.50 ± 1.40^a^	26.70 ± 1.70^a^	27.60 ± 1.38^a^	24.60 ± 1.27^b^	27.60 ± 1.29^a^
Internode length (cm)	4.00 ± 0.40^a^	4.20 ± 0.32^a^	4.00 ± 0.36^a^	3.80 ± 0.41^a^	4.50 ± 0.35^a^
Number of leaves	6.60 ± 0.70^a^	6.40 ± 0.52^a^	6.90 ± 0.57^a^	6.50 ± 0.53^a^	6.20 ± 0.63^a^
Leaf area (cm^2^)	25.70 ± 4.83^a^	26.10 ± 4.96^a^	27.30 ± 6.98^a^	25.90 ± 7.18^a^	21.90 ± 5.99^a^
Leaf inclination angle (°)	46.10 ± 2.98^d^	48.90 ± 5.48^cd^	53.30 ± 3.94^bc^	55.50 ± 3.59^b^	61.50 ± 4.76^a^
Petiole length (cm)	8.60 ± 0.64^c^	9.20 ± 0.67^b^	9.70 ± 0.75^b^	9.10 ± 0.61^bc^	10.80 ± 0.79 ^a^
Total root length (cm)	113.50 ± 22.40^c^	190.30 ± 20.10^a^	206.00 ± 32.40^a^	153.00 ± 19.10^b^	105.10 ± 11.80^c^
Leaves biomass (mg DM)	355.41 ± 40.79^a^	267.20 ± 33.86^c^	307.88 ± 30.08^b^	321.04 ± 29.77^ab^	208.75 ± 34.22^d^
Stem biomass (mg DM)	371.19 ± 14.63^b^	447.10 ± 16.48^a^	389.72 ± 12.28^b^	373.46 ± 11.03^b^	269.05 ± 10.78^c^
Roots biomass (mg DM)	45.93 ± 6.08^c^	61.15 ± 6.25^a^	54.01 ± 5.84^ab^	52.28 ± 6.51^b^	40.43 ± 5.86^c^
Shoot-to-root ratio (S/R DM)	15.82 ± 1.76^a^	11.68 ± 1.27^c^	12.92 ± 1.40^bc^	13.28 ± 1.27^b^	11.82 ± 1.64^c^
Stomatal traits (abaxial leaf surface)					
Stomatal width (µm)	21.13 ± 0.99^a^	18.82 ± 0.88^b^	17.16 ± 0.80^bc^	16.19 ± 0.76^c^	17.03 ± 0.80^c^
Stomatal length (µm)	25.44 ± 0.59^a^	23.15 ± 0.53^b^	22.05 ± 0.51^b^	18.50 ± 0.43^c^	18.95 ± 0.44^c^
Stomatal width/length ratio	0.83 ± 0.03^b^	0.81 ± 0.03^bc^	0.78 ± 0.02^c^	0.87 ± 0.03^a^	0.90 ± 0.03^a^
Pore width (µm)	3.22 ± 0.12^a^	2.13 ± 0.16^b^	2.09 ± 0.16^b^	2.08 ± 0.11^b^	2.23 ± 0.17^b^
Pore length (µm)	11.07 ± 0.96^a^	9.74 ± 0.32^b^	8.00 ± 0.45^c^	6.78 ± 0.26^d^	5.66 ± 0.44^e^
Stomatal pore area (µm^2^)	26.40 ± 3.30^a^	14.57 ± 2.90^b^	10.40 ± 2.10^c^	9.92 ± 2.00^c^	9.87 ± 2.00^c^
Stomatal density *S*_d_ (no. mm^–2^)	267.30 ± 20.89^b^	349.78 ± 12.82^a^	214.86 ± 17.48^c^	221.59 ± 24.16^c^	232.19 ± 14.11^c^
Pore area per leaf area (µm^2^ mm^–2^)	7056.63 ± 551.61^a^	5096.29 ± 186.73^b^	2234.54 ± 181.81^c^	2198.17 ± 239.65^c^	2291.68 ± 139.29^c^

The values are means of ten replicates ± SD. For the individual stomatal traits, a magnification of × 1000 or × 250 for *S*_d_ was used. The leaves DM relates to DM of all leaves of an individual plant (with petioles). The shoot DM in S/R ratio is the sum of the DM of the stem and leaves (with petioles) of an individual plant. Different letters (a–e) in the same row indicate significant differences between the treatments at *p* = 0.05 with a Tukey’s HSD test

*DM* dry mass

### Stomatal traits in the various lighting spectra

In response to increasing G light intensity, the plants shared a general tendency to contract their stomatal dimension (Table 2). The length of the stomatal apparatus of G10 and G20 plants decreased by nearly 9% and 13%, whilst in G30 and G40 plants, it was by 27% and 26%, respectively, relative to the control plants. In plants cultivated with 30% and 40% G light in the spectrum, a nearly 23% and 19% reduction in the width of the stomatal apparatus was noticed, whilst in G10 and G20, it was on average 11% and 19% lower than in the RB plants. Pore dimensions of individual stomata were also decreased under RGB treatment, compared to RB. Pore width was reduced by approximately one-third in all plants grown under the RGB spectrum, whilst the pore length was reduced by 12%, 28%, 39% and 49% in G10, G20, G30 and G40 plants, respectively. Thus, although the stomatal width/length ratio decreased in G10 and G20 plants by only 2% and 6%, their pore area was reduced by 45% and 61%, respectively. Moreover, the G30 and G40 plants possessed more circular stomata, with approximately 5% and 8% higher width/length ratios, respectively, than in C plants.

The stomatal density (*S*_d_) on the abaxial leaf surface decreased by about 20%, 17% and 13% in G20, G30 and G40 plants, respectively, but in G10, *S*_d_ increased by 31%, as compared to the control group. As expected, the pore area per leaf area (sum of pore area per leaf area) was not even and the leaves, which developed in the presence of green light, had a significantly lower pore area per leaf area than the RB-grown leaves, which means that leaves of plants grown under RGB possessed less and rounder stomata with much smaller pores.

### The effect of light quality on photosynthetic pigment level, PRI and anthocyanin accumulation

The decreased concentration of chlorophyll *a* was only detected in the G20 group, and only when compared to the G30 plants (Table 3). Overall, the spectrum did not have any influence on the Chl *b* level or the analysed Chl *a*/*b* ratio. Carotenoids were estimated as a pool, and the lowest content was also found in G20 (Table 3), but the differences amongst other plant groups were not significant. Consequently, no differences in the PRI between GX and C plants were noted (Table 3), indicating that there was no significant influence of light spectrum make-up on xanthophyll pigment composition. The PRI reflects short-term reversible changes in the epoxidation state of xanthophyll pigments, which are the major components of non-photochemical quenching (Sun et al. 2014) that translates into the changes in reflectance at 531 nm, relative to the reflectance at 570 nm.

**Table 3:** The abundance of photosynthetic pigments extracted with DMSO from 3-mm leaf discs, photochemical reflectance index (PRI), the accumulation of anthocyanins and soluble protein (SLP) content in tomato leaves under different light conditions

Parameter	Treatment				
	C	G10	G20	G30	G40
Chl *a* + *b* (mg g^–1^ FM)	3.20 ± 0.51^ab^	3.00 ± 0.42^ab^	2.80 ± 0.28^b^	3.40 ± 0.36^a^	3.00 ± 0.40^ab^
Chl* a* (mg g^–1^ FM)	2.60 ± 0.40^ab^	2.40 ± 0.32^ab^	2.20 ± 0.21^b^	2.60 ± 0.29^a^	2.40 ± 0.30^ab^
Chl *b* (mg g^–1^ FM)	0.66 ± 0.12^a^	0.64 ± 0.10^a^	0.62 ± 0.10^a^	0.72 ± 0.08^a^	0.68 ± 0.10^a^
Chl *a/b*	3.90 ± 0.31^a^	3.70 ± 0.17^a^	3.60 ± 0.37^a^	3.70 ± 0.20^a^	3.50 ± 0.20^a^
Carotenoids (mg g^–1^ FM)	0.54 ± 0.08^a^	0.51 ± 0.06^ab^	0.46 ± 0.04^b^	0.56 ± 0.06^a^	0.48 ± 0.06^ab^
PRI	0.038 ± 0.005^a^	0.039 ± 0.005^a^	0.039 ± 0.003^a^	0.036 ± 0.003^a^	0.040 ± 0.004^a^
Anthocyanins (AU g^–1^ FM)	1.80 ± 0.01^b^	2.00 ± 0.06^a^	1.70 ± 0.03^c^	1.10 ± 0.02^d^	0.70 ± 0.02^e^
Soluble leaf proteins (mg g^–1^ FM)	15.33 ± 0.91^ab^	16.28 ± 1.12^a^	14.52 ± 0.72^b^	12.94 ± 0.65^c^	11.30 ± 0.67^d^

The presented values are means of ten (or four for SLP) replicates ± SD. Different letters (a–e) in the same row indicate significant differences between treatments at *p* = 0.05 with a Tukey’s HSD test

*AU* arbitrary unit, *FM* fresh mass

It should be noted, however, that the addition of more than 10% of the G light to the combined spectrum of R and B light affected the anthocyanins level, the amount of which was progressively reduced by about 6% in G20 and about 60% in G40, as compared to C (Table 3).

### The effect of growth spectrum composition on the subsequent photosynthetic efficiency of PSII

**Fig. 2:**
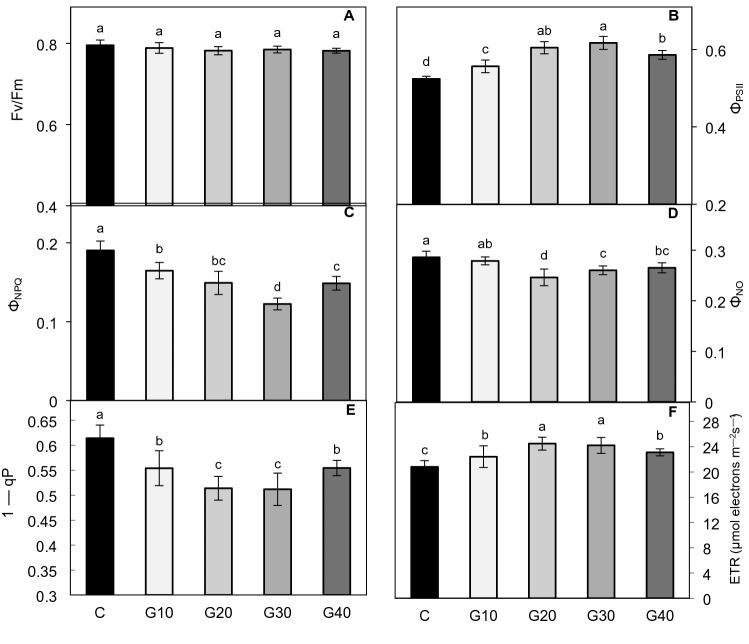
Effects of growth light quality on **A** maximum quantum yield of PSII photochemistry (Fv/Fm), **B** effective quantum yield of PSII photochemistry (*Φ*_PSII_), **C** quantum yield of regulated (*Φ*_NPQ_) and **D** non-regulated energy dissipation (*Φ*_NO_), **E** excitation pressure on PSII (1 − *qP*) and **F** electron transport rate (ETR). The analyses were carried out with 110 µmol m^–2^ s^–1^ (**A**–**D**) or 186 µmol m^–2^ s^–1^ (**E** and **F**) of blue (450 nm) actinic light. The values are means of six replicates ± SD. Different letters (*a*–*d*) indicate significant differences between treatments at *p* = 0.05 with a Tukey’s HSD test

The photosynthetic efficiency of PSII (Fv/Fm), as well as the ways in which the absorbed energy was used for photochemical (*Φ*_PSII_) and non-photochemical quenching (*Φ*_NPQ_, *Φ*_NO_), were estimated with a low intensity of blue AL (110 µmol m^–2^ s^–1^). No differences in the Fv/Fm values (0.782–0.796) between tomato groups were found (Fig. 2A). It appeared, however, that *Φ*_PSII_ was significantly higher in all GX groups, especially in G20 and G30 plants, as compared to C (Fig. 2B). The improvement in the photosynthetic efficiency showed an almost linear trend up to 30% of G light contribution in the spectrum. Additionally, decreased *Φ*_NPQ_ values in the G10–G40 groups (Fig. 2C), without any accompanying increases in *Φ*_NO,_ were also observed (Fig. 2D). Such observations have also been confirmed in the analyses of the electron transport rate and the excitation pressure in response to higher AL intensities (186 µmol m^–2^ s^–1^). Plants grown under the combined RGB spectrum presented higher ETR (Fig. 2F), whilst the 1 − *qP* on PSII was usually much lower in these plants than in C (Fig. 2E). A significant difference in 1 − *qP* values between G10 and C suggests that the substitution of as little as 10% of R light with G successfully alleviated the excitation pressure placed on PSII.

### G light quantum efficiency and in situ measurements

**Fig. 3:**
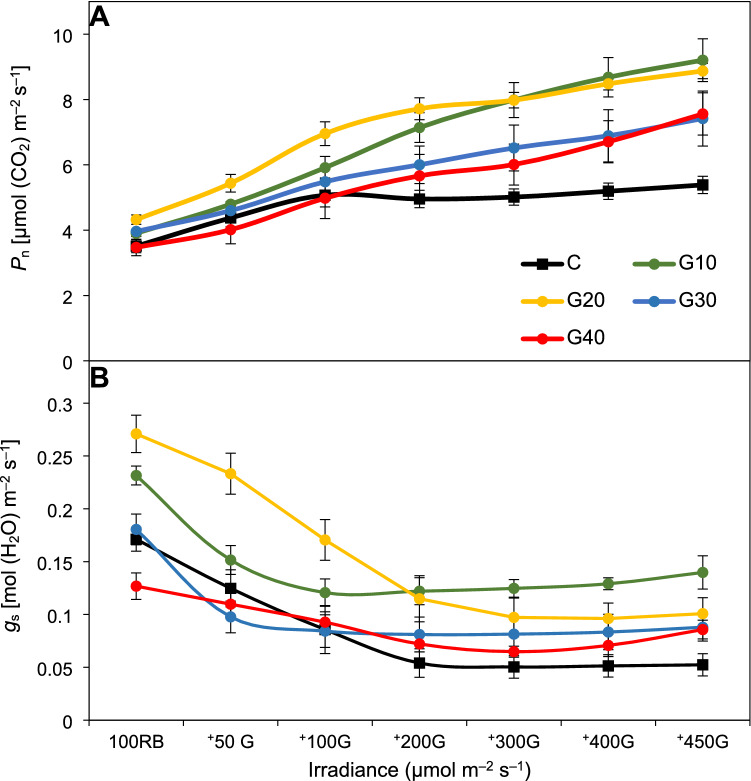
**A** Net photosynthetic rate (*P*_n_) and **B** stomatal conductance (*g*_s_) recorded in 2 × 3-cm transparent chamber in response to 0–550 μmol m^–2^ s^–1^ PPFD of constant 75 μmol m^–2^ s^–1^ of red (R, 627 nm) and 25 μmol m^–2^ s^–1^ of blue (B, 447 nm) light and variable doses of green light (G, 530 nm) (0, 50, 100, 200, 300, 400 and 450 μmol m^–2^ s^–1^) provided by SL 3500-C LED illuminator. The values are means of four replicates ± SD

The addition of low doses of G light (50 or 100 μmol m^–2^ s^–1^, + 50G or + 100G, respectively) to a constant background of RB increased *P*_n_ values (Fig. 3A), indicating that tomato plants, regardless of the previous growth light quality, were able to utilize the additional G light for carbon fixation. However, higher doses of G light (> 100 μmol m^–2^ s^–1^) only led to enhanced assimilation rates in GX plants, whilst the CO_2_ fixation in the control group remained at a roughly constant level. Finally, under the 450 μmol m^–2^ s^–1^ of additional G light, the net photosynthesis increased by 54%, 87%, 105%, 118% and 135% in C, G30, G20, G40 and G10 (Fig. 3A). At the same time, the inclusion of G light to the combined RB background noticeably reduced the leaf stomatal conductance. The tested groups, however, exhibited differences in stomatal response starting from the initial step of illumination (Fig. 3B). Consequently, the *g*_s_ value in the G20 plants was much higher under the 100 μmol m^–2^ s^–1^ of RB light, as well as under additional the 50 or 100 μmol m^–2^ s^–1^ of G light in comparison to C or the remaining GX groups. Nevertheless, the stomatal conductance values in the plants previously grown under the RGB spectrum were usually higher than those in C in response to G light (Fig. 3B).

The analysed quantum yield of green light (530 nm, *Φ*_530_) demonstrated that all plants used G light to drive photosynthesis, although with different efficiencies. Plants that were grown previously under RGB spectrum showed a significantly higher ability to utilize a higher photon flux of G light for CO_2_ fixation than those grown solely under RB. Consequently, a stepwise increase in G light intensity resulted in a significant enhancement of carbon dioxide assimilation, especially in G10, which presented the highest value of *Φ*_530_ (*Φ*_530_ = 0.012 µmol (CO_2_) µmol^–1^ photons), as compared to C (*Φ*_530_ = 0.003 µmol (CO_2_) µmol^–1^ photons) (Table 4).

**Table 4:** The quantum yield of green light (530 nm) (*Φ*_530_) was based on a non-linear regression equation (below) of a fitted non-rectangular hyperbola using the non-linear fitting procedure (NLIN) for CO_2_ fixation rate per absorbed green light unit

Parameter	Treatment				
	C	G10	G20	G30	G40
*Φ*_530_ [µmol (CO_2_) µmol^–1^ photons]	0.003 ± 0.0005^d^	0.012 ± 0.0015^a^	0.009 ± 0.0012^b^	0.007 ± 0.0009^c^	0.008 ± 0.0016^bc^
Non-linear regression equation	y = − 0.1417 + 2.0312 × log10(x)	y = − 10.5811 + 7.1603 × log10(x)	y = − 6.7195 + 5.6809 × log10(x)	y = − 4.5596 + 4.2532 × log10(x)	y = − 6.8792 + 5.1225 × log10(x)
Absorptance of 530 nm (%)	75.82 ± 3.86^a^	74.59 ± 4.28 ^a^	74.86 ± 4.52^a^	77.16 ± 2.16^a^	74.40 ± 4.35^a^

The absorptance of green light (530 nm) was based on the leaf optical properties measured with spectroradiometer GL SPECTIS 5.0 Touch integrated with LI-1800-12S sphere (LI-COR Inc.) with a tungsten halogen lamp emitting a broad-spectrum of 380–2500 nm. Different letters (a–d) in the same row indicate significant differences between treatments at *p* = 0.05 with a Tukey’s HSD test

**Fig. 4:**
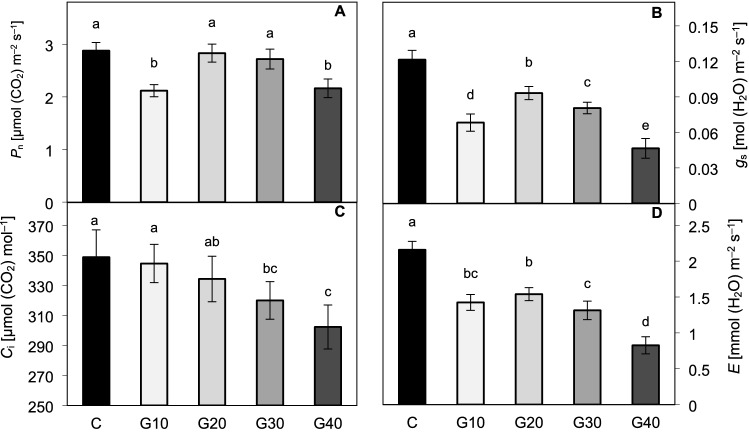
Gas exchange parameters measured in situ in the 2 × 3-cm transparent chamber. **A** Net photosynthetic rate (*P*_n_), **B** stomatal conductance (*g*_s_), **C** intercellular carbon dioxide concentration (*C*_i_) and **D** transpiration rate (*E*). The values are means of four replicates ± SD. Different letters (*a*–*e*) indicate significant differences between treatments at *p* = 0.05 with a Tukey’s HSD test

**Fig. 5:**
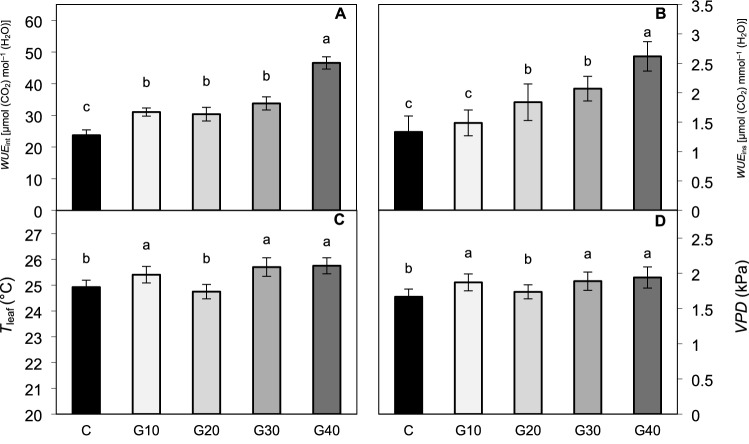
Gas exchange parameters measured in situ in the 2 × 3-cm transparent chamber. **A** intrinsic water-use efficiency (*P*_n_/*g*_s_) (*WUE*_int_), **B** instantaneous water-use efficiency (*P*_n_/*E*) (*WUE*_ins_), **C** leaf temperature (*T*_leaf_) and **D** vapour pressure deficit (*VPD*). The values are means of four replicates ± SD. Different letters (*a*–*c*) indicate significant differences between treatments at *p* = 0.05 with a Tukey’s HSD test

At the same time, in the experiment performed under chamber-specific low light intensity, the G10 and G40 plants exhibited a lowered net photosynthetic rate (*P*_n_) as compared to C (Fig. 4A), considered to be a consequence of decreased stomatal conductance (*g*_s_) (Fig. 4B), that confined CO_2_ influx into the leaves (*r* = 0.95 for *g*_s_ to *C*_i_ correlation). However, this explanation turned out to be valid only for G40 plants, presenting significantly lowered *C*_i_ (Fig. 4C) along with decreased *g*_s_ and *P*_n_. Overall, *P*_n_ value has shown to be less correlated to *C*_i_ (*r* = 0.80) than to *g*_s_ (*r* = 0.91). Other factors such as mesophyll conductance could also be considered to influence *P*_n_ (Bian et al. 2019). Analysed transpiration rate (*E*) (Fig. 4D) was positively correlated to *g*_s_ value (*r* = 0.96) and lowered under all RGB treatments compared to RB treatment. Consequently, by analysing *WUE*_int_ (Fig. 5A) and *WUE*_ins_ (Fig. 5B), it appeared that plants grown under RGB light tended to utilize water more efficiently per CO_2_ assimilated than C plants. The *WUE*_int_ values in G10–G30 plants were nearly 1.3–1.4-times higher than in C and reached twice as high a level in G40 plants.

Vapour pressure deficit (*VPD*) and leaf temperature *(T*_leaf_) are considered as sensitive indicators of plant stress (O’Carrigan et al. 2014). In this study, *T*_leaf_ (Fig. 5C) and *VPD* (Fig. 5D) were affected by the spectrum composition and showed a negative correlation to *g*_s_ (*r* = − 0.77 and *r* = − 0.94, respectively) and *E* (*r* = − 0.75 and *r* = − 0.92, respectively). Consequently, replacement of red light with the green light in the spectrum, except for G20 treatment, lead to an increase of *T*_leaf_ by 2%, 3% and 3.5% and of *VPD* by 12%, 13% and 16% in G10, G30 and G40 plants, respectively.

### The effect of light quality on soluble protein content

**Fig. 6:**
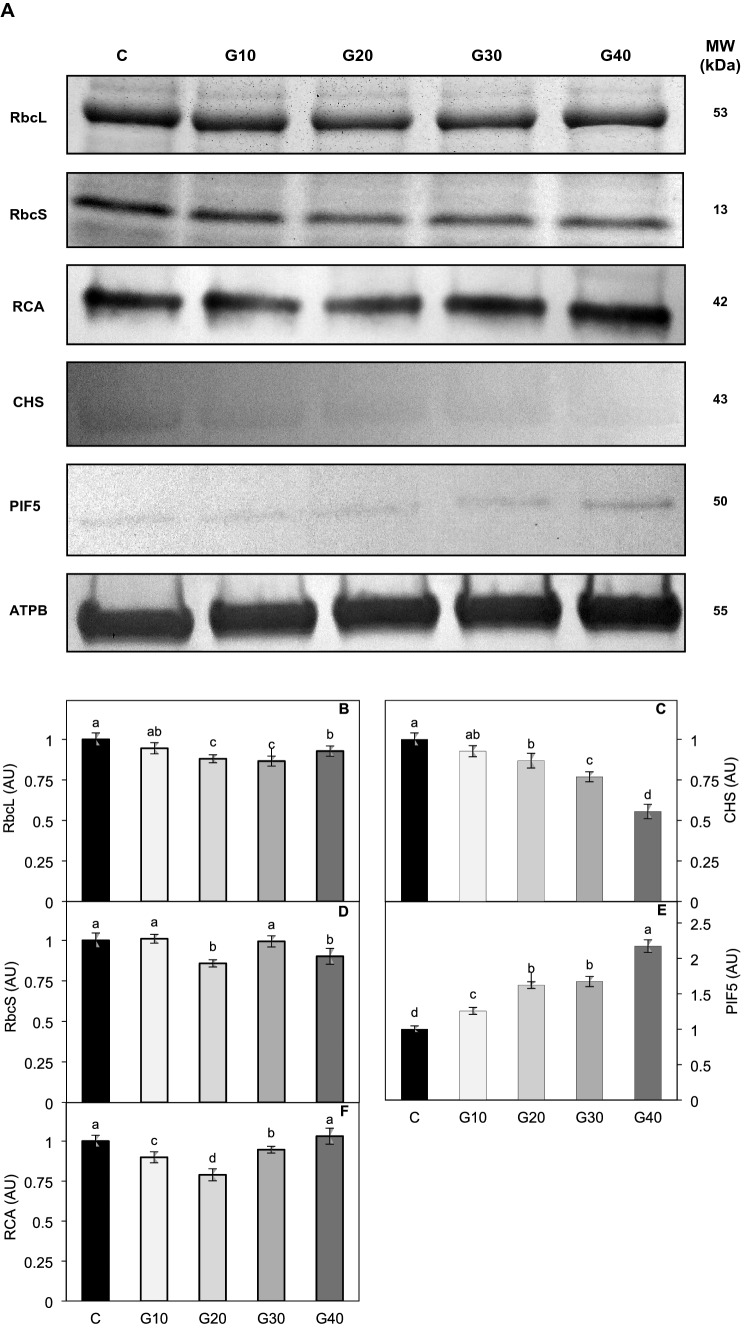
Changes in the amount of large (RbcL) and small (RbcS) subunit of Rubisco, Rubisco activase (RCA), chalcone synthase (CHS) and phytochrome interacting factor 5 (PIF5) in leaves of *Solanum lycopersicum* L. cv. Malinowy Ozarowski grown under different LED illumination. ATPB is the loading control. **A** Coomassie staining (RbcL and RbcS) and immunoblotting (RCA, CHS, PIF5 and ATPB) were performed using extracts containing leaf proteins. To visualize RbcL, RbcS, RCA and ATPB, the aliquots containing 5 μg of total protein were used, whilst for PIF5 and CHS, the aliquots containing 60 µg of total protein were loaded to each lane in the gel. The quantification of the Coomassie-stained protein bands of **B** RbcL and **D** RbcS, as well as DAB-stained **F** RCA, **C** CHS and **E** PIF5, was carried out using ImageJ densitometric analysis. The relative amount of protein of interest was assessed based on the assumption that the densitometric value recorder for C plants is unity (100%). The values are means of four replicates ± SD. Different letters (*a*–*d*) indicate significant differences between treatments at *p* = 0.05 with a Tukey’s HSD test. *AU* arbitrary unit, *MW* molecular weight

The concentration of soluble leaf proteins in plants grown under RGB with 30% and 40% of G light was lower than in control by 16% and 26%, respectively (Table 3). Meanwhile, the lowest level of the Rubisco large subunit (RbcL) was detected in G20 and G30 plants (Fig. 6A, B), whilst of the Rubisco small subunit (RbcS) in G20 and G40 groups (Fig. 6A, D). The immunoblot analysis demonstrated that the changes in the Rubisco content in GX plants roughly correlated with the Rubisco activase (RCA) level (Fig. 6A, F). Furthermore, consistent with an increasing contribution of G light in the spectrum, there was a simultaneous decrease in the CHS enzyme level in G20, G30 and G40 plants compared to the C plants (Fig. 6A, C). Then, to evaluate a possible correlation of CHS and anthocyanin levels with the SAS response, the content of PIF5 was also determined. As expected, the PIF5 abundance in the plants grown under the RGB spectrum (Fig. 6A, E) was significantly higher than that in the C plants and gradually increased with the progressive replacement of R light by G light in the growth spectrum. Consequently, the PIF5 level in G40 plants was over twofold higher than in the control group.

## Discussion

### The influence of RGB spectrum on tomato plant morphology

Meng et al. (2020) reported that the incremental substitution of G radiation with R radiation, in the absence of B light, did not influence any of the parameters measured. The authors documented, however, that under low B radiation (20 μmol m^–2^ s^–1^), G radiation increased the shoot fresh mass and leaf number. In the present study, the applied spectrum modification altered the plant morphology and partitioning of biomass. Substituting G light with R at the same B light PPFD in G10, G20 and G40 plants decreased the dry biomass of leaves, although it did not affect the leaf number or its area. Moreover, the reduced leaf biomass observed, except for G40, was associated with increased biomass accumulation and the length of roots. This could indicate that the tomato responds to G photons by prioritizing biomass partitioned into the root with diminished partitioning into leaf lamina. At the same time, substituting G light up to 20% did not reduce the plants’ height, internode length or stem dry biomass. Furthermore, the addition of 10% of green light in place of red light even increased DM of the stem. However, the application of 30% G light in the spectrum already reduced growth dynamics, whilst the plants grown under the spectrum with 40% G light presented significantly reduced dry biomass of the aboveground structures. Overall, the replacement of R light with G light in the spectrum in each dose lowered significantly the shoot-to-root dry mass ratio. Mickens et al. (2019) documented that red pak choi plants grown under a spectrum supplemented with G light appeared to reduce biomass partitioning into leaf lamina, whilst He et al. (2020) documented that G-treated potato plants had a lower rate of photosynthate allocation in organs, as compared to W, R or B light treatment.

### Shade-avoidance syndrome (SAS) under RGB light

In this work, we revealed that tomato plants grown under low light conditions (100 μmol m^−2^ s^−1^) with up to about 30% of green photons only displayed limited morphological traits that were recognized to be a SAS response (Yang and Li 2017), such as the increased length of petioles and the inclination angle of leaves. However, under the spectrum predominantly occupied by G light (35% R, 40% G, 25% B) besides the mentioned traits, the plants presented a significantly reduced dry biomass. Moreover, it has been previously shown (Wang et al. 2015) that a SAS response is closely related to phytochrome interacting factors (PIFs) level. Indeed, an increased PIF5 abundance was detected in tomato plants grown under the RGB spectrum, especially in G40 plants. The increment of the PIF5 (and other PIFs) level has been predominantly assigned to the progressive reduction of R light contribution in the growth spectrum (Liu et al. 2015). However, the natural shade is a combination of the reduction in the R/FR ratio, as well as the reduction in B and UV irradiance, and the increased G/B ratio (Yang and Li 2017). Thus, we speculate that progressive substitution of R with the G light potentiates the SAS response, especially when the G light contribution in the spectrum exceeds that occupied by B. Such speculation is based on the discussed differences of SAS manifestation between G30 (G/B = 1.2) and G40 (G/B = 1.6) plants (Table 1). Additionally, Fankhauser and Batschauer (2016) have proposed that an increase in the G/B ratio causes similar stem elongation to a decreased R/FR ratio and different G/B ratios affect the formation of the CRY-PIF (mainly PIF5) complex.

### The influence of spectrum quality on CHS level and anthocyanin accumulation

PIF5 is also a transcriptional repressor of red light-induced up-regulation of anthocyanin biosynthetic genes (Liu et al. 2015). Additionally, Kitazaki et al. (2018) have found that the G light influences the repression of anthocyanin biosynthesis genes, whilst Tran et al. (2021) documented that W and G light decreased anthocyanin content compared to B light. Our results support such observations since we have found a negative correlation between the increased PIF5 level in RGB plants and the anthocyanin level (*r* = − 0.87). Furthermore, the reduced anthocyanins accumulation observed has been accompanied by diminished CHS levels in GX plants (*r* = 0.94), which were negatively correlated with PIF5 levels (*r* = − 0.97). Meanwhile, high CHS and anthocyanins levels were detected in RB60 plants (see Appendix Fig. S5; Table S2) that were grown under 60 μmol m^–2^ s^–1^ PPFD and a spectrum analogous to that applied for G40 plants, but devoid of the green component (35R:25B). The most noticeable feature of the RB60 plants was, however, the low level of PIF5, which was opposite to that found in G40 plants (Fig. S5). These results, as well as the previous reports (Bouly et al. 2007; Zhang and Folta 2012; Herbel et al. 2013; Kitazaki et al. 2018), suggest that the opposing effects of G and B light, but not a reduced intensity of R light, were predominantly responsible for the increased SAS-related PIF5 content, along with a reduced content of CHS and anthocyanins in GX plants. Such a finding indicates that reduced R light intensity might be just as necessary, but not sufficient, for negative regulation of anthocyanin biosynthesis in tomato plants.

### Photosynthetic pigment content under G-enriched spectrum

We essentially did not observe greater differences in both chlorophyll and carotenoid concentrations amongst the tomato groups, which is evidence that G light exerted no negative influence on the pigment accumulation when applied with a constant B light background. Chen et al. (2020) also proved that the substitution of red with green LEDs in the presence of a fixed proportion of blue LEDs did not alter the concentration of chlorophylls and carotenoids, as compared to RB light treatment.

### Implications of spectral composition for subsequent light utilization for photosynthesis

In agreement with previous reports (Bian et al. 2018a; Hamdani et al. 2019), we have demonstrated that growth-light composition influenced the subsequent ability to effectively utilize light for photosynthesis. The replacement of R light with a G in growth spectrum significantly reduced the excitation pressure on PSII and *Φ*_NPQ_, and improved the rate of electron transport. Liu et al. (2017) suggested that the addition of G light to the spectrum affects the subsequent light absorption utilized for photosynthesis, whilst Trojak and Skowron (2021) showed that tomato plants grown under monochromatic G or mixed RGB light presented a decreased NPQ rate, as compared to monochromatic red or blue light. Additionally, Yousef et al. (2021) showed that the best performance of the photosynthetic apparatus was observed under a mixture of R and B light (R7: B3) or a mixture of R, G and B light (R3: G2: B5).

Moreover, plants grown under the RGB spectrum were able to utilize additional G light (530 nm) more efficiently than RB plants. He et al. (2021) stated that photosynthetic light-use efficiency and photosynthetic gas exchange rate are affected by the quality of the LED lighting. However, the increased CO_2_ fixation of RGB plants cannot be explained by either the increased photosynthetic pigments content or the leaf area change. It could be explained, at least partly, by the specific absorption of the green part of the spectrum (530–550 nm) by anthocyanins (Laby et al. 2000; Zheng et al. 2020). Increased accumulation of anthocyanins in RB plants reduced the amount of the G light successfully absorbed by photosynthetically active pigments, thereby decreasing the quantum yield of green light, as compared to GX plants. Another explanation is a common pattern in the regulatory action of G light on cell growth and density of their packing in the leaf mesophyll or level of photosynthetic pigments in a single chloroplast (Golovatskaya and Karnachuk 2015). Consequently, these adaptations would influence the distribution of green light within the leaf, thereby affecting the effectiveness of its absorption and utilization (Liu and van Iersel 2021). Therefore, despite no differences in absorptance of 530 nm were observed amongst treatments (Table 4), a more thorough analysis of leaf mesostructure under different light spectra is required to explain that phenomenon.

In our experiment, we also observed a stomatal closure response induced by G light within all the plant groups, which effectively counteracted the effects of the constant amount of RB light (100 μmol m^–2^ s^–1^) in the spectrum. Frechilla et al. (2000) also documented that G light was able to reverse the previous B light-induced stomatal opening response. Whilst the possible explanation for the observed discrepancies between the assimilation rate and stomatal opening following G light illumination (RB + G_curve_) is that the stomatal opening is regulated predominantly by a photosynthesis-independent mechanism based on the G/B light antagonism associated with B and G light sensitive receptors, such as CRYs (Battle and Jones 2020), rather than a decrease in the intercellular CO_2_ concentration*.*

### The influence of light quality on actual gas exchange and stomatal traits

The spectrum composition seems to be crucial to maintain the effective carbon dioxide fixation since both low (10%) and high (40%) G light contribution in the RB spectrum decreased *P*_n_, compared to C, G20 and G30. At the same time, we observed that G light supplementation, in all the contributions applied, reduced stomatal conductance and transpiration rate, increasing water-use efficiency. Talbott et al. (2006) suggested that G light-stimulated reversal of the B light-induced stomata opening might prevent excessive leaf water loss. Additionally, Bian et al. (2019) documented that adding G light to the RB spectrum decreases the stomatal conductance, which enhanced the intrinsic and instantaneous water-use efficiency. Moreover, similar to Liu et al. (2017) we found that *VPD* and *T*_leaf_ presented an opposite trend to *g*_s_. O’Carrigan et al. (2014) documented that G light treatment of tomato leaves led to a significant increase of *VPD* and *T*_leaf_ along with highly decreased *g*_s_ and *E* compared to monochromatic R, B or W light and that such response was related to modified geometry of guard cells. Thus, we propose that, under the RB spectrum, a crucial factor determining the low water-use efficiency is the significant level of B light in the spectrum (25%), along with the absence of G light, as documented by Claypool and Lieth (2021). Such an assumption was confirmed in the G10 plants, as the significant improvement of *WUE*_int_ has been noticed with as little as 10% substitution of R with G light.

However, it was unclear whether reduced *g*_s_ was caused by the direct effect of R light substitution with G light on stomatal opening or by structural changes and the number of stomata per leaf area. It has been previously reported by Macedo et al. (2011) and O’Carrigan et al. (2014) that G light influences stomata formation. Consistent with this, we found that the major response of tomato plants to low doses of G light (10%) was a decreased pore area of individual stomata, along with increased stomatal density, whereas in response to higher G light contribution (20‒40%), the plants developed leaves with fewer stomata and had reduced pore areas. Pore area, however, is dynamically adjusted by changes in pore width, whilst pore length is rather rigid during the opening and closure of stomata (Fanourakis et al. 2015). Meanwhile, the present results show that reduction of pore area in response to the increasing percentage of G light was not equally influenced by contracting width and length of individual pore. We found that pore width contraction was reduced in a similar way in all RGB treatments, thus it was presumably caused by an opposing effect of G light on B light-driven stomatal opening. In the case of pore length, however, it was progressively reduced along with increasing G light contribution in the spectrum as a result of reduced stomatal length (*r* = 0.96). Thus, results indicate that differences in stomatal conductance and transpiration rate between light treatments lie not only in the functional properties of stomata in response to light quality but also in anatomical components of stomata and their number influenced by spectrum composition.

### Enzymes of CO_2_ assimilation under RGB spectrum

The Rubisco content is strictly related to the light conditions used for plant growth. Moreover, as Rubisco is the predominant protein in leaves of C3 plants contributing up to 50% to the soluble leaf proteins (Feller et al. 2008), modification of Rubisco content also influences the content of SLP. In fact, we observed that 30% and 40% contribution of G light in place of R light in the spectrum lowered the SLP level, and observed that plants, which were grown under RGB light, share a tendency to decrease both Rubisco and RCA contents. An exception to this was a control-like level of RCA in G40 plants that might be interpreted as an attempt to overcome the effects of reduced stomatal conductance, which impeded the CO_2_ influx. The action of G light is associated with a decreased expression of the Rubisco subunit genes (*rbcS* and *rbcL*) (Su et al. 2014; Wu et al. 2014) and the Rubisco activase gene (*rca*) (Su et al. 2014). The influence of the spectrum composition on Rubisco and RCA level was, however, less pronounced than its effect on actual CO_2_ assimilation, and the level of enzymes did not correspond to the net photosynthetic rate measured in situ. Interestingly, the analysed RB60 plants, despite the lowered PPFD, presented an almost C-like level of both Rubisco subunits (Fig. S5) and consequently C-like level of SLP (Table S2). Thus, it is reasonable to assume that the Rubisco level is rather related to the constant intensity of blue light applied in all sets of growth chambers than to a decreasing R light intensity. Therefore, the observed reduction in Rubisco subunits content in RGB plants might be related to the negative impact of G light on the Rubisco level; however, the effect of spectrum composition on RCA remains largely unclear.

## Conclusion

Under low light conditions, supplementation of the RGB spectrum with up to 30% of G light exerted mostly beneficial effects, whilst the addition of 40% of G light was undesirable as it potentiated the SAS response and significantly reduced the dry biomass of plants. Replacing G light with R stimulated the elongation of petioles and altered the pattern of biomass accumulation; under RGB light, plants transport more assimilates to the roots rather than to leaves, as compared to those grown under the RB-LEDs. Additionally, the increased PIF5 level and the subsequently restricted CHS and anthocyanins levels were noticed in response to additional G light. The RGB spectrum increased the photosynthetic yield with less energy being dissipated by the NPQ mechanisms, and alleviated the photoreduction of the plastoquinone Q_A_ pool and substantially reduced the stomatal conductance and transpiration due to the decreased size and number of the leaf stomata and the possible involvement of green–blue light antagonism, which leads to an improvement in the plant water-use efficiency. Finally, the results of the quantum yield for CO_2_ fixation demonstrated that G light was able to drive the photosynthesis efficiently, whilst the effectiveness of CO_2_ fixation within G light depended upon light conditions applied during plant growth. Thus, the results confirmed the prediction that the quality of the growth spectrum influences the photosynthetic utilization of available radiant energy and the mechanisms of photoprotection.

## Supplementary Information

Below is the link to the electronic supplementary material.

Supplementary file1 (DOCX 49507 kb)

## Data Availability

The datasets generated during and/or analysed during the current study are available from the corresponding author on reasonable request.

## Abbreviations

AU: Arbitrary unit
B: Blue (light)
Chl *a*(*b*): Chlorophyll *a*(*b*)
CHS: Chalcone synthase
DAT: Days after transplanting
DM: Dry mass
*E*: Transpiration rate
ETR: Electron transport rate
Fv/Fm: Maximal quantum yield of photosystem II (PSII) photochemistry
FM: Fresh mass
G: Green (light)
*g*_s_: Stomatal conductance
LED: Light-emitting diode
NPQ: Regulated non-photochemical quenching
PIF: Phytochrome interacting factor
*P*_n_: Net Photosynthetic rate
PPFD: Photosynthetic photon flux density
PRI: Photochemical reflectance index
R: Red (light)
RbcL/RbcS: The large/small subunit of ribulose-1,5-bisphosphate carboxylase/oxygenase
RCA: Ribulose-1,5-bisphosphate carboxylase/oxygenase activase
SAS: Shade-avoidance syndrome
*S*_d_: Stomatal density
SLP: Soluble leaf proteins
*T*_leaf_: Temperature of leaf measured with thermocouple
*VPD*: Vapour pressure deficit
*WUE*_ins_: Instantaneous water-use efficiency
*WUE*_int_: Intrinsic water-use efficiency
*Φ*_530_: The quantum yield of the 530 nm monochromatic light
*Φ*_NO_: The quantum yield of non-regulated energy dissipation
*Φ*_NPQ_: The quantum yield of regulated energy dissipation
*Φ*_PSII_: The effective quantum yield of PSII photochemistry
