# Supplementary material for: Effects of partial replacement of red by green light in the growth spectrum on photomorphogenesis and photosynthesis in tomato plants
Source: Photosynth Res. 2021 Sep 27;151(3):295–312. doi: 10.1007/s11120-021-00879-3 (PMC8940809; doi:10.1007/s11120-021-00879-3)
Supplement: Supplementary file 1 — Supplementary file1 (DOCX 49507 kb) [file 11120_2021_879_MOESM1_ESM.docx]

**Appendix**

**Fig. S1** Sunlight spectral composition, in the range of 400–700 nm, on a sunny day at noon (Kielce, Poland, 50°52'N 20°37'E), recorded with a spectroradiometer **GL SPECTIS 5.0 Touch** (**GL Optic Lichtmesstechnik GmbH, Weilheim/Teck, Germany)**

| \|  \| \| --- \| |  |  |  |  |  |  |  |  |  |  |
| --- | --- | --- | --- | --- | --- | --- | --- | --- | --- | --- | --- |
|  |  |  |  |  |  |  |  |  |  |  |
|  |  |  |  |  |  |  |  |  |  |  |
|  |  |  |  |  |  |  |  |  |  |  |
|  |  |  |  |  |  |  |  |  |  |  |
|  |  |  |  |  |  |  |  |  |  |  |
|  |  |  |  |  |  |  |  |  |  |  |
|  |  |  |  |  |  |  |  |  |  |  |
|  |  |  |  |  |  |  |  |  |  |  |
|  |  |  |  |  |  |  |  |  |  |  |
|  |  |  |  |  |  |  |  |  |  |  |
|  |  |  |  |  |  |  |  |  |  |  |
|  |  |  |  |  |  |  |  |  |  |  |
|  |  |  |  |  |  |  |  |  |  |  |
|  |  |  |  |  |  |  |  |  |  |  |
|  |  |  |  |  |  |  |  |  |  |  |
|  |  |  |  |  |  |  |  |  |  |  |
|  |  |  |  |  |  |  |  |  |  |  |
|  |  |  |  |  |  |  |  |  |  |  |
|  |  |  |  |  |  |  |  |  |  |  |
|  |  |  |  |  |  |  |  |  |  |  |
|  |  |  |  |  |  |  |  |  |  |  |
|  |  |  |  |  |  |  |  |  |  |  |
|  |  |  |  |  |  |  |  |  |  |  |
|  |  |  |  |  |  |  |  |  |  |  |
|  |  |  |  |  |  |  |  |  |  |  |
|  |  |  |  |  |  |  |  |  |  |  |
|  |  |  |  |  |  |  |  |  |  |  |
|  |  |  |  |  |  |  |  |  |  |  |
|  |  |  |  |  |  |  |  |  |  |  |
|  |  |  |  |  |  |  |  |  |  |  |

**Fig. S2** The light spectra of growth chambers were recorded with a spectroradiometer at six locations at the level of the apical bud and averaged. Plants of C and G10–G40 groups were grown under 100 µmol m^–2^ s^–1^ PPFD, while RB60 plants were grown under 60 µmol m^–2^ s^–1^. C states for the control plants (75R:25B). RB60 plants were grown under RB light (35R:25B), analogous to the spectrum applied for G40 plants but devoid of the green component. The rest of the plants (G10–G40) were grown under the RGB spectrum provided by progressive replacing from 10% (G10) to 40% (G40) of R light with an equal amount of G light. B light intensity was kept constant at 25 µmol m^–2^ s^–1^ in all chambers

**A**


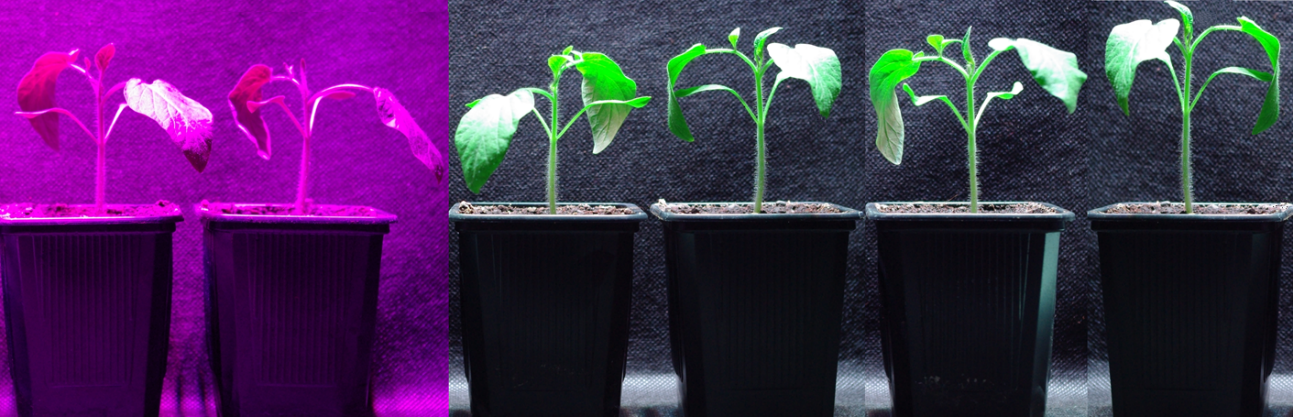


5 cm

**C RB60 G10 G20 G30 G40**

**B**


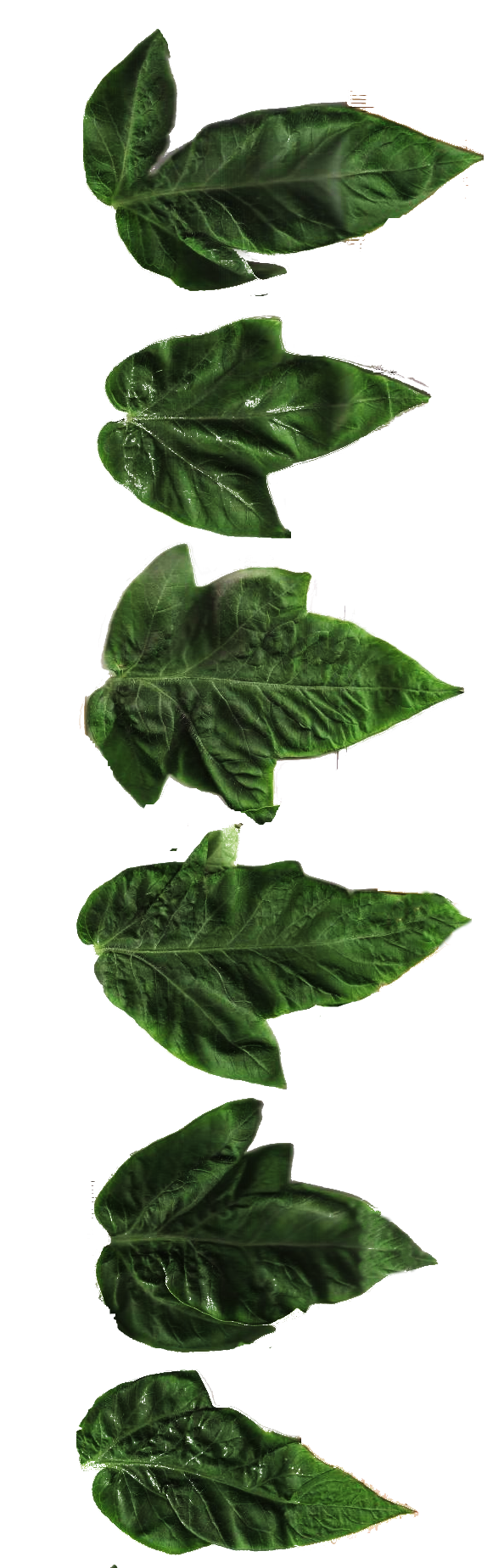


1 cm

**C RB60 G10 G20 G30 G40**

**C**


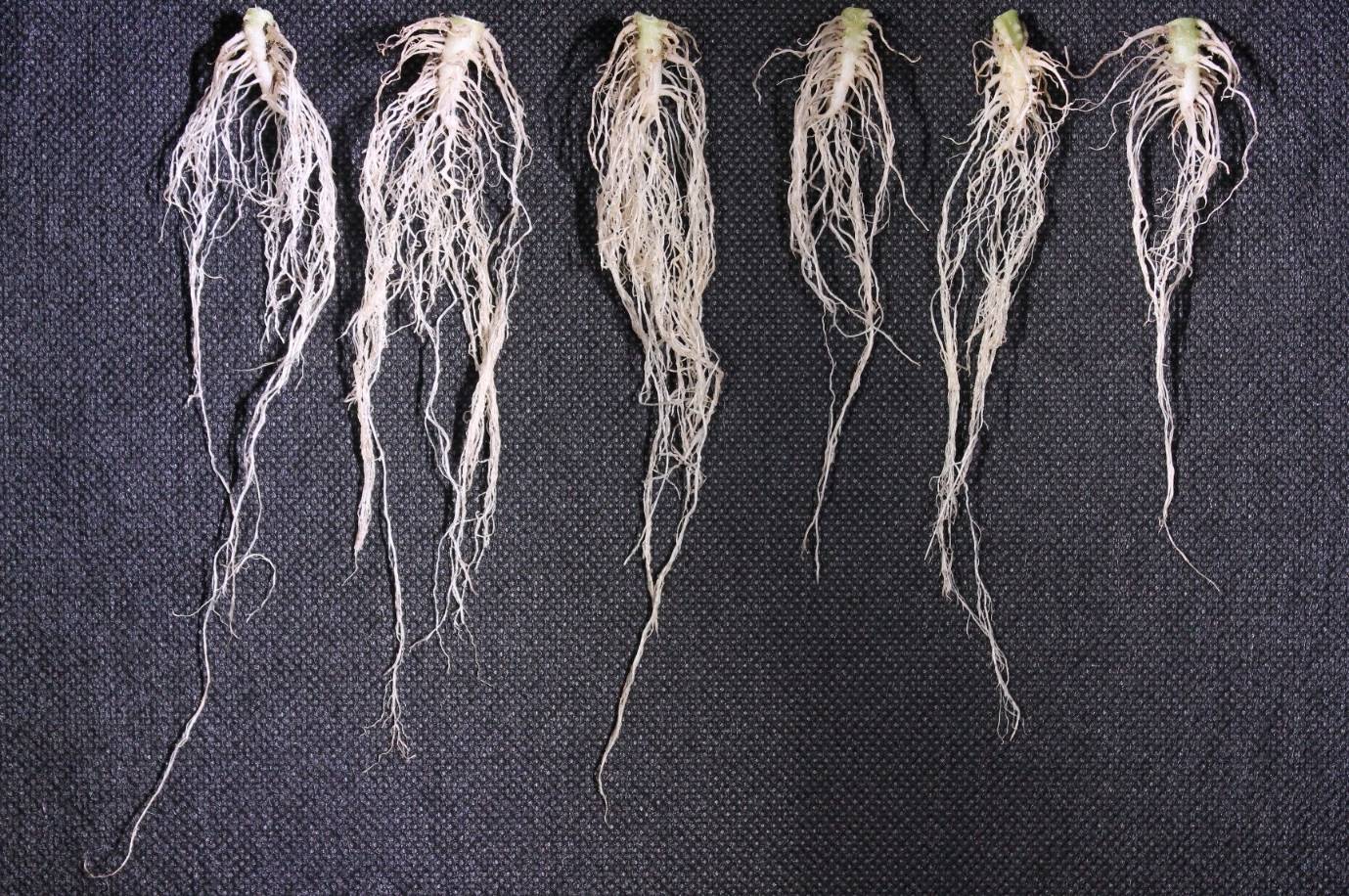


**G10 G20 G30 G40 C RB60**

5 cm

**Fig. S3** Effects of different light qualities on the growth and morphological appearances of tomato plants. (**A**) 6-days-after-transplanting (DAT) plants; (**B**) leaves of 24-DAT plants; (**C**) roots of 24-DAT plants. The C group was used as a control group for G10–G40 plants, while the RB60 was used as an additional control for G40 plants to show the influence of G light addition to the RB spectrum on morphological traits

**G30**


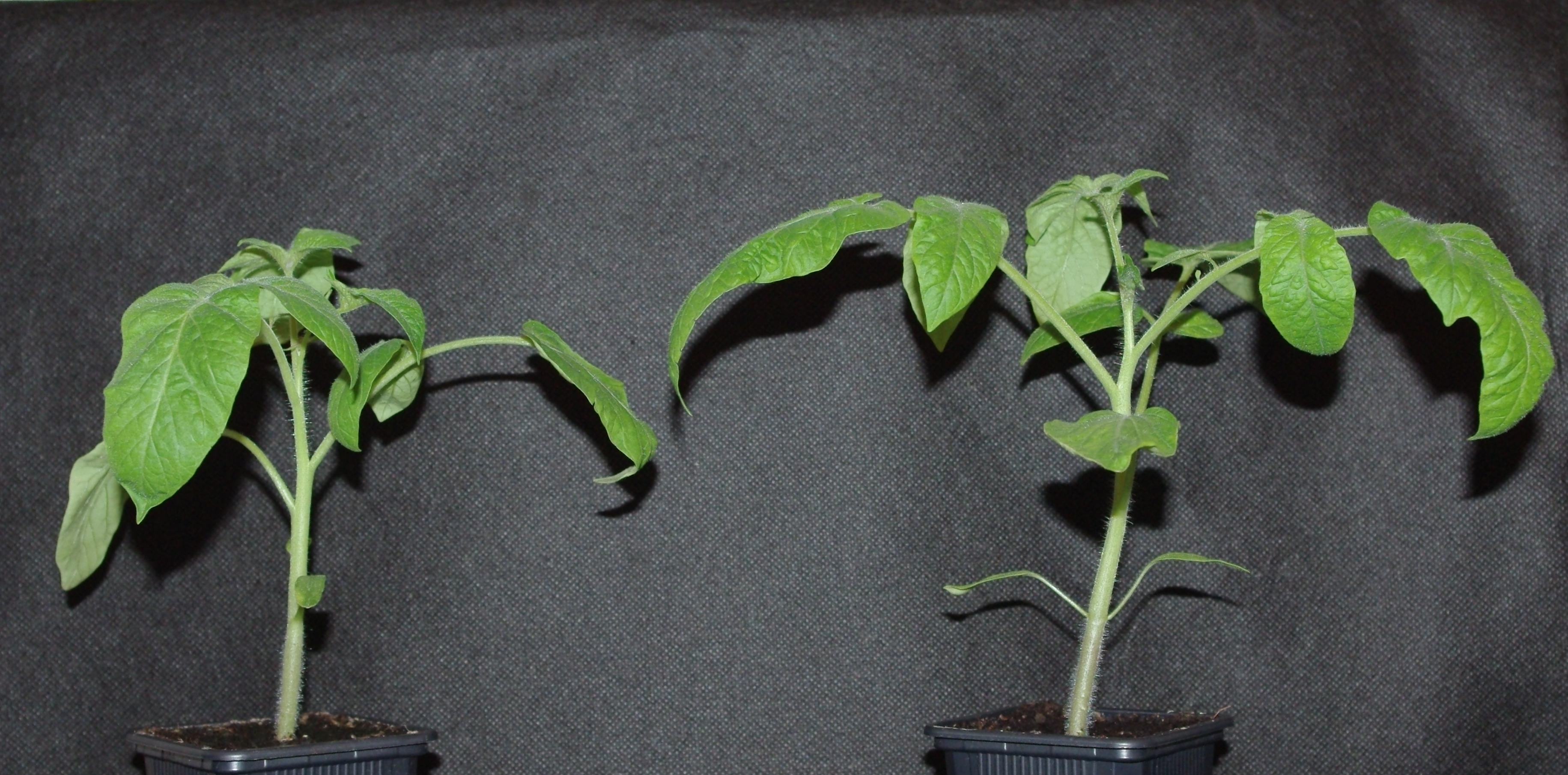

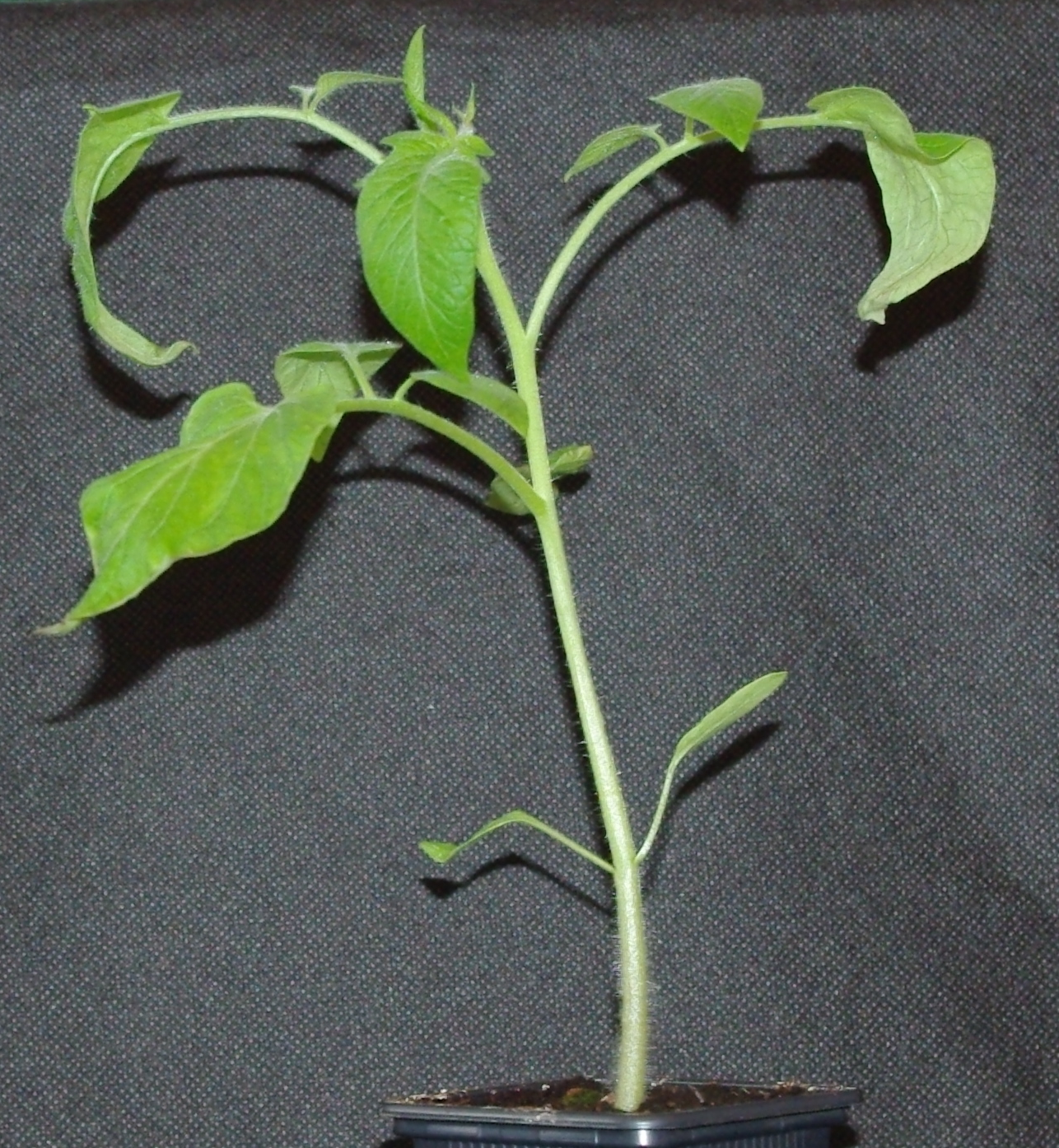

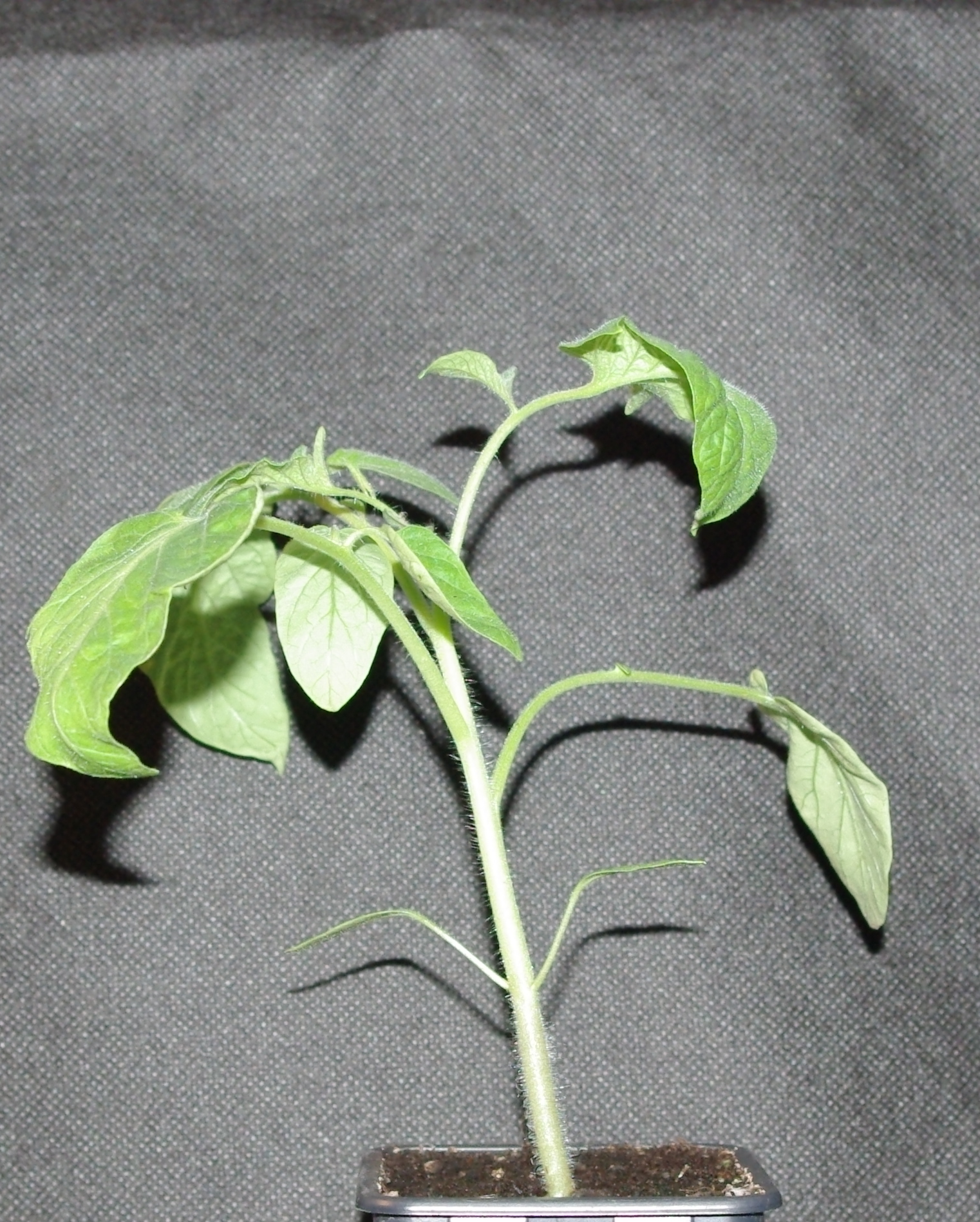


**G20**

**G30**

**G40**

**G10**

**C (RB)**

**Fig. S4** Effects of different light qualities on the growth and morphological appearances of 24-days-after-transplanting (DAT) tomato plants. The C plants were used as a control group for G10–G40 to show the influence of G light addition to the RB spectrum on photomorphogenesis. The photo of the G40 plant presented also the leaf inclination angle measurement (**A**), which is the angle of the leaf above the horizontal with the base of the petiole at the vertex

**MW**

**(kDa)**

**RbcL 53**

**RbcS 13**

**RCA 42**

**PIF5 50**

**CHS 43**

**ATPB 55**


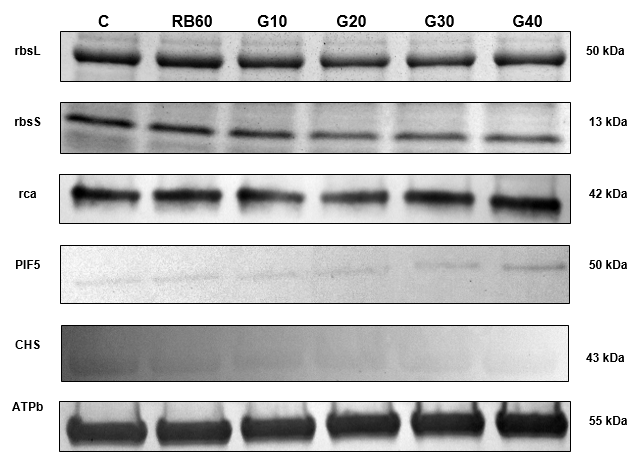


**Fig. S5** Changes in the amount of large (RbcL) and small (RbcS) subunit of Rubisco, Rubisco activase (RCA), phytochrome interacting factor 5 (PIF5) and chalcone synthase (CHS) in leaves of *Solanum lycopersicum* L. cv. Malinowy Ozarowski grown under different LED illumination. ATPB is the loading control. To visualize RbcL, RbcS, RCA and ATPB the aliquots containing 5 μg of total leaf protein were used, while for PIF5 and CHS, the aliquots containing 60 µg of total leaf protein were loaded to each lane in the gel. Bands represent the Coomassie-stained protein of RbcL and RbcS, as well as DAB-stained RCA, PIF5, CHS and ATPB, analysed with four repetitions. The C plants were used as a control group for G10–G40 plants, while RB60 plants served as an additional control for G40 to show the influence of additional G light added to the RB spectrum on the protein level. MW – molecular weight

**Table S1** Description of the spectral composition used in the study within the RB60 group and the ratio of photon flux integral (µmol m^–2^ s^–1^) of R and FR (R/FR), G and B (G/B), R and B (R/B) radiation. The light spectrum was recorded with a spectroradiometer at six locations at the level of the apical bud and averaged. RB60 plants were grown under 60 µmol m^–2^ s^–1^ of RB light (35R:25B), analogous to the spectrum applied for G40 plants but devoid of the green component

| Treatment | Spectral characteristics of lighting treatments | | | | | |  |
| --- | --- | --- | --- | --- | --- | --- | --- |
|  | % R**^a^** | % G | % B | FR**^b^** | R/FR | G/B | R/B |
| RB60 | 35 | 0 | 25 | 0.02 | 1908.0 | – | 1.4 |

^a^ Percentage of R (601−700 nm), G (501−600 nm) and B (401−500 nm) radiation of total (60 µmol m^–2^ s^–1^) PPFD (400−700 nm)

^b^ Photon flux integral of FR (701−750 nm) radiation in µmol m^–2^ s^–1^

**Table S2** Anthocyanins accumulation and soluble protein (SLP) content in tomato leaves under different light conditions. The presented values are means of ten (or four for SLP) replicates ± SD. Different letters (a–f) indicate significant differences between treatments at *p*=0.05 with a Tukey’s HSD test. AU – arbitrary unit; FM – fresh mass

| Parameter | Treatment | | | | | |
| --- | --- | --- | --- | --- | --- | --- |
|  | C | RB60 | G10 | G20 | G30 | G40 |
| Anthocyanins (AU g^−1^ FM) | 1.80 ± 0.01^c^ | 2.10 ± 0.02^a^ | 2.00 ± 0.06^b^ | 1.70 ± 0.03^d^ | 1.10 ± 0.02^e^ | 0.70 ± 0.02^f^ |
| Soluble leaf proteins (mg g^–1^ FM) | 15.33 ± 0.91^ab^ | 15.78 ± 0.87^a^ | 16.28 ± 1.12^a^ | 14.52 ± 0.72^b^ | 12.94 ± 0.65^c^ | 11.30 ± 0.67^d^ |
